# Engineering Sugar Utilization and Microbial Tolerance toward Lignocellulose Conversion

**DOI:** 10.3389/fbioe.2015.00017

**Published:** 2015-02-18

**Authors:** Lizbeth M. Nieves, Larry A. Panyon, Xuan Wang

**Affiliations:** ^1^School of Life Sciences, Arizona State University, Tempe, AZ, USA

**Keywords:** synthetic biology, metabolic engineering, lignocellulose, xylose, furan aldehydes

## Abstract

Production of fuels and chemicals through a fermentation-based manufacturing process that uses renewable feedstock such as lignocellulosic biomass is a desirable alternative to petrochemicals. Although it is still in its infancy, synthetic biology offers great potential to overcome the challenges associated with lignocellulose conversion. In this review, we will summarize the identification and optimization of synthetic biological parts used to enhance the utilization of lignocellulose-derived sugars and to increase the biocatalyst tolerance for lignocellulose-derived fermentation inhibitors. We will also discuss the ongoing efforts and future applications of synthetic integrated biological systems used to improve lignocellulose conversion.

## Introduction

One of the daunting challenges faced by the modern world is our unsustainable dependence on petroleum as the primary source for transportation fuels and many chemical products including solvents, fertilizers, pesticides, and plastics (Service, 2007). To fulfill future societal needs, we have to find a sustainable supply of energy and chemicals. Synthetic biology has emerged as a young discipline with the great potential to construct a novel biological system to produce fuels and chemicals from renewable sources in a cost-effective manner, thus ultimately achieving energy self-sufficiency independent of petroleum. We will apply the synthetic biology definition of “the design and construction of new biological components, such as enzymes, genetic circuits, and cells, or the redesign of existing biological systems” throughout this review (Keasling, 2008). The engineered biological systems created by synthetic biology include enzymes with new functions, genetic circuits, and engineered cells with unique specifications (Cameron et al., 2014; Way et al., 2014). In many cases, the ultimate goal is to rationally manipulate organisms to facilitate novel functions, which do not exist in nature (Cameron et al., 2014; Way et al., 2014). Thus far, synthetic biology has contributed to many fields such as bio-based production (Keasling, 2008; Jarboe et al., 2010), tissue and plant engineering (Bacchus et al., 2012; Moses et al., 2013; Xu et al., 2013; Trantidou et al., 2014), and cell-free synthesis (Lee and Kim, 2013).

**Figure 1:**
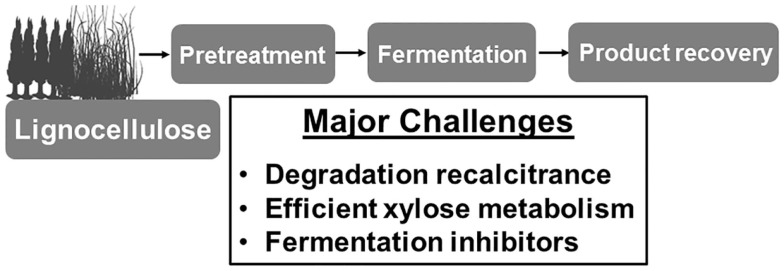
**Challenges of lignocellulose conversion**. Lignocellulose regularly needs pretreatment to release its sugar components for biocatalysts to make fuels and chemicals. This is a sustainable approach to reduce our dependence on petroleum and to prevent carbon dioxide emission. At least three major challenges remain to be solved for a cost-effective lignocellulose conversion.

Plant biomass (lignocellulose) represents arguably the most important renewable feedstock on the planet. Lignocellulose is a complex matrix of various polysaccharides, phenolic polymers, and proteins that are present in the cell walls of woody plants (Saha, 2003; Girio et al., 2010). Conversion of non-food plant biomass, especially agricultural residues such as corn stover and sugarcane bagasse, avoids the many concerns about the production of fuels and chemicals derived from food sources (Lynd, 1990). Additionally, non-food-based biofuels offer greater cost reduction in the longer term (Lynd, 1990). For numerous types of agricultural residues, the sugar content is comparable to corn (Saha, 2003). However, the conversion of these sugars from agricultural residues to fuels and chemicals in a cost-effective manner still remains challenging. There are at least three major challenges to be solved before lignocellulose bioconversion becomes financially feasible (Figure 1). First, in contrast to starch, which is easily degraded into fermentable sugar monomers, sugars in lignocellulose are locked into very stable polymeric structures including cellulose and hemicellulose (Saha, 2003; Girio et al., 2010). These polymers are designed by nature to resist deconstruction (Alvira et al., 2010). The crystalline-like fibers of cellulose are encased in a covalently linked mesh of lignin and hemicellulose. Cellulose (30–40% of biomass dry weight) is composed of only d-glucose linked by β-1,4 glycosidic bonds while a mixture of pentoses, especially d-xylose, and hexoses comprises the main component of hemicellulose (20–40% of biomass dry weight) (Saha, 2003). Lignin is not the saccharides polymer but a complex polymer of aromatic alcohols. Different types of lignocellulosic biomass vary in the composition of cellulose, hemicellulose, and lignin (Saha, 2003). Chemical pretreatment processes are commonly required for lignocellulose conversion. Steam pretreatment with dilute mineral acids is an efficient approach to depolymerize hemicellulose into sugar monomers and to increase the accessibility of cellulase enzymes to degrade cellulose (Saha, 2003; Sousa et al., 2009; Alvira et al., 2010). After pretreatment and cellulase digestion, most of the sugars in agricultural waste will be released into the broth and thus ready to be converted into fuels and chemicals if a suitable biocatalyst is applied. The cost of cellulase enzymes is currently still prohibitive to wide application of lignocellulose conversion. Continuing efforts of synthetic biologists from academic and industrial labs are improving cellulase enzymes or enzyme complexes aiming to develop catalysts that are cost-effective enough to be suitable for commercialization. The recent advancements in cellulases have been extensively reviewed (Elkins et al., 2010; Garvey et al., 2013; Hasunuma et al., 2013; Bommarius et al., 2014) and therefore are not the scope of this review. Second, one of the major carbohydrates in the typical lignocellulosic biomass is d-xylose, a five-carbon aldose, which is difficult for many microbes to metabolize. For instance, common ethanol-producing industrial microbes such as *Saccharomyces cerevisiae* and *Zymomonas mobilis*, do not natively metabolize xylose (Saha, 2003). Although some microbes such as *Escherichia coli* and *Klebsiella pneumonia* have the native xylose metabolic pathway, it is not efficient and is commonly repressed by the presence of glucose (Saha, 2003). Third, side products that hinder cell growth and fermentation such as furfural, 5-hydroxymethylfurfural, formate, acetate, and soluble lignin products are formed during common chemical pretreatment processes (Saha, 2003; Mills et al., 2009). For example, furfural (dehydration product of pentose sugars) is widely regarded as one of the most potent inhibitors (Mills et al., 2009; Geddes et al., 2010a, 2011). It can completely inhibit cellular growth at low concentrations (Zaldivar et al., 1999; Liu and Blaschek, 2010). The concentration of furfural is correlated with the toxicity of dilute acid hydrolyzates (Martinez et al., 2000). Overliming to pH 10 with Ca(OH)_2_ or active carbon filter reduces the level of furfural and toxicity, but increases the process complexity and operational cost, thus reducing economic viability (Martinez et al., 2000). There has been a growing interest to engineer industrially related strains to be more resistant to these inhibitors (Wang et al., 2012a,b; Zheng et al., 2012; Geddes et al., 2014; Xiao and Zhao, 2014). For example, beneficial genetic traits to increase host tolerance of furan aldehydes have been identified (Taherzadeh et al., 2000; Liu et al., 2004, 2005, 2008; Gorsich et al., 2006; Petersson et al., 2006; Almeida et al., 2008; Geddes et al., 2014; Glebes et al., 2014a,b; Luhe et al., 2014), knowledge about toxicity mechanisms has been accumulated (Lin et al., 2009a; Miller et al., 2009a,b; Ma and Liu, 2010; Glebes et al., 2014a,b), and thus the integrated synthetic detoxification systems have been constructed and proven effective in different biocatalysts (Wang et al., 2013).

Despite government incentives and mandates, these grand challenges have prohibited the commercialization of lignocellulose conversion into fuels and chemicals at low cost (Sheridan, 2013). Until now, most efforts for lignocellulose conversion have been devoted to microbial ethanol production. By pathway engineering and metabolic engineering, the microbial hosts can extend their metabolism to produce valuable chemicals other than ethanol from lignocellulose. This review focuses on engineering new biological components by synthetic biology to improve lignocellulose conversion. The past efforts, current status, and future challenges will be discussed.

## Genetic Improvement of Utilization and Transport of Monosaccharides Derived from Lignocellulose

Hydrolysis of hemicellulose and cellulose into five- and six-carbon sugars by pretreatments provides the mixture of sugars. Microorganisms tend to selectively utilize a preferred sugar, usually d-glucose, by a regulation mechanism called catabolite repression. Synthetic biology has the potential to re-design microbial biology to simultaneously use d-glucose and other pentoses efficiently. Lignocellulosic raw materials commonly contain much higher amounts of d-xylose compared to other pentoses, and therefore, improving xylose fermentation has become a priority (Girio et al., 2010). Xylose degradation is not universal for all microbes in spite of being the most abundant monosaccharide in hemicellulose. At the current stage, most related research still uses the trial-and-error approach to accelerate xylose transport and xylose metabolism. A more quantitative understanding of sugar catabolism is necessary before synthetic biologists are able to predict and design a biological system that efficiently transports and metabolizes sugars.

**Figure 2:**
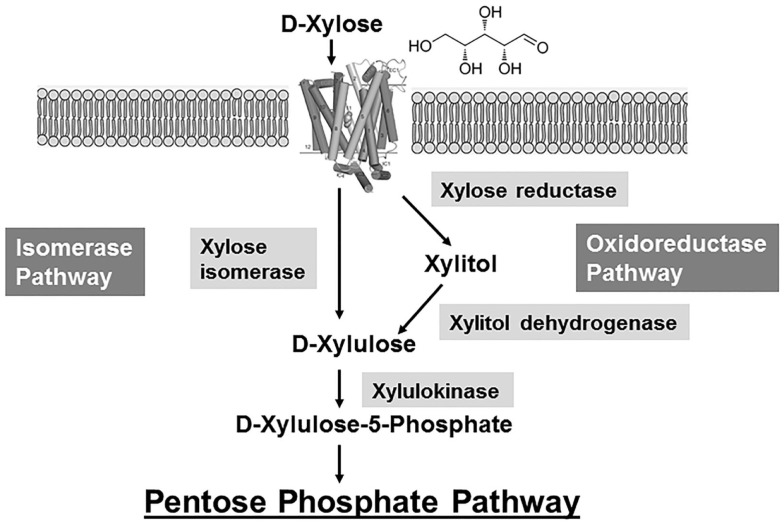
**Two metabolic pathways of d-xylose metabolism**. Xylose is transported into cells and then it is either isomerized by xylose isomerase in some bacteria or reduced to xylitol by xylose reductase in some fungi. Xylitol is oxidized to xylulose and then phosphorylated to form xylulose-5-phosphate by xylulokinase. Xylulose-5-phosphate enters the pentose phosphate pathway for further degradation. The isomerase pathway avoids the production of xylitol.

There are two major metabolic pathways to catabolize xylose: xylose isomerase pathway and oxidoreductase pathway used by bacteria and fungi, respectively (Figure 2). These pathways have been constructed and optimized in industrial biocatalysts such as *S. cerevisiae* and *Z. mobilis*, which cannot natively metabolize xylose. There are comprehensive reviews that excellently summarized this research topic (Jeffries and Jin, 2004; Chu and Lee, 2007; Matsushika et al., 2009; Young et al., 2010; Cai et al., 2012; Kim et al., 2013). Here, we only briefly review some of important past efforts. The xylose oxidoreductase pathway is commonly used by some ascomycetous yeasts such as *Pichia stipitis* (Figure 2). Although the *S. cerevisiae* chromosome has genes encoding xylose reductase, xylitol dehydrogenase, and xylulokinase, their native expression level is too low to support cellular growth when using xylose as the sole carbon source (Yang and Jeffries, 1997; Richard et al., 2000; Traff et al., 2002; Toivari et al., 2004). Anaerobic xylose fermentation by *S. cerevisiae* was first demonstrated by heterologous expression of *XYL1* (Rizzi et al., 1988) and *XYL2* (Rizzi et al., 1989) genes encoding xylose reductase and xylitol dehydrogenase from *P. stipitis* (Kotter et al., 1990; Tantirungkij et al., 1994). However, the xylitol is accumulated as a significant side product when genes *XYL1* and *XYL2* are overexpressed in the recombinant *S. cerevisiae*, which lowers the ethanol yield. The accumulation of xylitol is likely due to the cofactor imbalance of the first two steps in the oxidoreductase pathway (Figure 2). NADPH is the preferred cofactor for xylose reductase to reduce xylose, while NAD is used by xylitol dehydrogenase to oxidize xylitol, resulting in the formation of xylulose (Figure 2). Unlike many bacteria, *S. cerevisiae* lacks pyridine nucleotide transhydrogenases, which catalyze the conversion between these two reducing cofactors, NADPH and NADH (Nissen et al., 2001). Therefore, this imbalance of cofactors caused by these two reactions will eventually lead to slow kinetics for xylose degradation and xylitol accumulation. Although overexpression of the xylose reductase and xylitol dehydrogenase genes has been shown to enable xylose metabolism in recombinant *S. cerevisiae* strains, overexpression of the xylulokinase gene is often required to create a complete functional heterologous pathway and to further reduce xylitol production (Ho et al., 1998; Jin et al., 2005; Bettiga et al., 2008). One successful example of engineering an efficient xylose-metabolizing yeast is the recombinant *Saccharomyces* sp. strain 1400(pLNH32) (Ho et al., 1998). In this strain, the *P. stipitis* xylose reductase, *P. stipitis* xylitol dehydrogenase, and *S. cerevisiae* xylulokinase genes under the control of the strong native glycolytic promoters were cloned into the plasmid pLNH32 to achieve high expression level. The aerobic conversion of xylose to ethanol has relatively high titer (23 g/L), yield (~0.45 g ethanol/g xylose, theoretic yield is ~0.5 g ethanol/g xylose for ethanol fermentation), and productivity (4 g/L/h) in a complex medium (Ho et al., 1998). Further improvements of ethanol titer and yields in several xylose-fermenting industrial yeast strains such as TMB 3400 and 424A(LNF-ST) have been achieved by utilizing the heterologous xylose oxidoreductase pathway and other genetic modifications that enhance the downstream pentose phosphate pathway (Matsushika et al., 2009). This demonstrates the potential of the xylose oxidoreductase pathway to improve xylose metabolism.

The xylose isomerase pathway, dominantly used by many bacteria including *E. coli* and *Bacillus subtilis*, has also been constructed in *S. cerevisiae* strains. In this pathway, xylose is directly converted to xylulose through a one-step reaction catalyzed by xylose isomerase or other aldose isomerases (Figure 2). This pathway does not involve xylitol formation and it does not require a reducing cofactor. However, this isomerization reaction thermodynamically favors xylose over xylulose at equilibrium (Jeffries, 1983), which requires an alternative driving force such as efficient downstream reactions to promote the equilibrium moving toward the formation of xylulose (Figure 2). In addition, it has been shown that the expression of functional bacterial xylose isomerase genes often result in inefficient enzymatic activities and thus low xylose utilization (Sarthy et al., 1987; Gardonyi and Hahn-Hagerdal, 2003). The unsuccessful heterologous expression is probably due to the protein misfolding and post-transcriptional modification. Even though the successful synthesis of active xylose isomerases derived from different microbes including thermophilic bacterium *Thermus thermophiles* (Walfridsson et al., 1996), *Piromyces* sp.E2 (Kuyper et al., 2003), *Orpinomyces* (Madhavan et al., 2009), and *Clostridium phytofermentans* (Brat et al., 2009) has been achieved in *S. cerevisiae* at high levels, the rate of growth on xylose was still poor. It is possible that further optimization is needed to increase metabolic flux of downstream reactions, especially the pentose phosphate pathway. Ethanol yield is often higher in these recombinant *S. cerevisiae* using the xylose isomerase pathway than those using the heterologous xylose oxidoreductase pathway because xylitol production is avoided. However, the titer and productivity of *S. cerevisiae* using the xylose isomerase pathway are very low. The *Piromyces* sp. xylose isomerase has been extensively engineered to increase catalytic efficiency, and the *S. cerevisiae* BY4741-S1 derivatives expressing this mutant enzyme improved both its aerobic growth rate and ethanol production (Lee et al., 2012). However, in terms of xylose utilization and ethanol production, these optimized recombinant *S. cerevisiae* strains still perform more poorly with a final ethanol titer lower than 4 g/L. The heterologous xylose isomerase pathway has also been successfully constructed in other biocatalysts such as *Z. mobilis*, a bacterium notable for its bioethanol-producing capabilities, which has been used as a natural fermentative agent in alcoholic beverage production (Skotnicki et al., 1983). Similar to *S. cerevisiae*, *Z. mobilis* cannot metabolize xylose, which limits its application in lignocellulose conversion. In addition, *Z. mobilis* metabolizes glucose into pyruvate using the Entner–Doudoroff pathway instead of glycolysis (Embden–Meyerhof–Parnas pathway) and then converts pyruvate into ethanol and CO_2_ (Conway, 1992). Even with the successful expression of the xylose isomerase and xylulokinase genes from *Xanthomonas campestris* or *Klebsiella pneumoniae*, *Z. mobilis* was still unable to grow using xylose as the sole carbon source (Liu et al., 1988; Feldmann et al., 1992). Interestingly, in addition to overexpression of the xylose isomerase and xylulokinase genes, overexpression of the transaldolase and transketolase genes (the main enzymes in the pentose phosphate pathway) resulted in a recombinant *Z. mobilis* with a functional xylose metabolism (Zhang et al., 1995). The resulting strain CP4 (pZB5) is able to convert xylose to ethanol with a higher titer (11 g/L) and yield (0.44 g/g xylose) compared to recombinant *S. cerevisiae* using the xylose isomerase pathway (Zhang et al., 1995). This excellent work strongly suggests a high flux of downstream metabolic reactions such as the pentose phosphate pathway is required for a functional xylose catabolism using the xylose isomerase pathway (Figure 2). A high performance of xylose to ethanol conversion using a bacterial xylose isomerase pathway has been achieved in a wild-type *E. coli* strain (ATCC9637) after extensive metabolic engineering and adaptive laboratory evolution (Jarboe et al., 2007). The recombinant *E. coli* strain LY180 uses the native xylose isomerase pathway and the *Z. mobilis* ethanol-producing pathway to achieve the efficient conversion of xylose to ethanol with a high titer (45 g/L after 48 h) and yield (0.48 g/g xylose) using mineral salts medium (Miller et al., 2009b; Yomano et al., 2009). These successful examples of engineering *Z. mobilis* and *E. coli* suggest that the bacterial xylose isomerase pathway has the potential for efficient xylose conversion when the metabolic flux in downstream pathways is efficient.

Another challenge for the conversion of sugars derived from lignocellulose is the sequential metabolism of sugar mixtures, a phenomenon called catabolite repression. d-glucose represses the utilization of other sugars such as xylose in many industrial catalysts, thus impeding the rapid and complete utilization of sugar mixtures during fermentation. The mechanism of glucose repression is very complex and involves multiple levels of regulation. For example, *E. coli* has complex glucose repression mechanisms mainly through cyclic AMP, cyclic AMP-binding protein and enzymes of the phosphotransferase system (Kim et al., 2010). There are also other mechanisms involving the inhibition of transport of alternative sugars and a dual transcriptional regulator called Cra (Ramseier, 1996). Strains with the relaxed glucose repression should be able to simultaneously use a heterogeneous sugar mixture. However, genetic perturbation of glucose repression components can disrupt regular glucose metabolism and result in decreased glucose metabolism. It is challenging to engineer a biocatalyst with relaxed glucose repression while keeping a high glucose utilization rate. There are different engineering strategies developed to improve sugar co-utilization (Yomano et al., 2009; Chiang et al., 2013). In a recombinant *E. coli* strain, a combinatory engineering strategy has achieved efficient co-utilization of glucose and xylose (30 g/L for each) in 16 h (Chiang et al., 2013). This genetic engineering strategy includes (1) deletion of *ptsG* (the glucose permease in phosphotransferase system) to release catabolite repression; (2) overexpression of a glucose transporter from *Z. mobilis* to restore glucose transport and metabolism; (3) overexpression of genes *rpiA*, *tktA*, *rpe*, and *talB* to increase pentose phosphate pathway. Recently, a completely different approach to decrease glucose repression has been developed (Galazka et al., 2010; Ha et al., 2011). Cellodextrins are glucose polymers of varying length (two or more glucose monomers) resulting from degradation of cellulose. Wild-type *S. cerevisiae* cannot assimilate cellodextrin because it lacks both the cellodextrin transporter and β-glucosidase capable of hydrolyzing cellodextrin into glucose. By integrating efficient transporters, the complemented hydrolytic enzymes for cellodextrin and the xylose oxidoreductase pathway (Figure 2) into *S. cerevisiae*, this recombinant *S. cerevisiae* strain is able to simultaneously consume cellodextrin and xylose probably because the glucose concentration is never high enough to induce the catabolite repression phenotype (Ha et al., 2011). It is plausible that intracellular hydrolysis of cellodextrin minimizes glucose repression of xylose fermentation allowing this co-consumption (Galazka et al., 2010; Ha et al., 2011). This novel strategy has the potential to enable efficient co-utilization of sugar mixtures derived from lignocellulose.

Successful lignocellulose conversion requires efficient transport of the mixture of sugars into the cells. The transport of xylose is less efficient than the transport of glucose and often inhibited by d-glucose, which suggests xylose transport is a limiting factor for lignocellulose conversion (Jeffries and Jin, 2004; Luo et al., 2014). Overexpression of homologous and heterologous sugar transporters enables recombinant strains to transport xylose, but have very limited positive effect on xylose fermentation and growth (Weierstall et al., 1999; Hamacher et al., 2002; Gardonyi et al., 2003; Sedlak and Ho, 2004; Saloheimo et al., 2007; Hector et al., 2008; Runquist et al., 2009). To improve xylose transporters, the substrate affinities for xylose of different yeast hexose transporters were altered and selected through mutagenesis and screening approaches (Young et al., 2012, 2014; Farwick et al., 2014). These efforts identified regions and motifs of the hexose transporters as the engineering targets for reprograming transporter properties (Farwick et al., 2014; Young et al., 2014). However, whether the transport of xylose is the limiting factor for xylose fermentation requires more characterization. Theoretically, xylose uptake becomes a limiting step only when the rate of xylose fermentation is higher than xylose uptake (Cai et al., 2012). The wild-type *S. cerevisiae* CEN.PK2-1C with its native hexose transporter Hxt was reported to be able to take up 0.14 g xylose/h/g dry cell weight in the presence of 50 mM xylose, which exceeds the xylose consumption rate in most recombinant *S. cerevisiae* strains (Hamacher et al., 2002; Cai et al., 2012). Without optimization of sugar transporters, engineered yeast strains already achieved relatively high performance of xylose fermentation using native hexose sugar transporters for xylose uptake (Ho et al., 1998; Sonderegger et al., 2004). The potential beneficial effect of these improved xylose transporters in the recombinant yeast strains with high xylose metabolism remains to be tested.

## Engineering Biocatalysts Resistant to Lignocellulose Inhibitors

Pretreatments such as dilute acid at elevated temperature are effective for the hydrolysis of pentose polymers in hemicellulose and also increase the access of cellulase enzymes to cellulose fibers. However, the fermentation of the resulting syrups, called hydrolyzates, is hindered by minor reaction products such as furan aldehydes including furfural and 5-hydroxymethylfurfural (5-HMF), organic acids, and phenolic compounds (Saha, 2003). Furfural and 5-HMF are formed by the dehydration of sugars (pentoses and hexoses, respectively) during pretreatment and more furfural than 5-HMF is present in most hemicellulose hydrolyzates (Saha, 2003; Geddes et al., 2010a,b, 2013). Furfural is of particular importance as a fermentation inhibitor because of its abundance and toxicity (Saha, 2003; Almeida et al., 2009; Mills et al., 2009; Geddes et al., 2010b, 2011). Furfural is more toxic than 5-HMF to industrial catalysts such as *E. coli* and *S. cerevisiae* (Zaldivar et al., 1999; Gorsich et al., 2006). In model studies with various hydrolyzate inhibitors, furfural was unique in potentiating the toxicity of other compounds (Zaldivar et al., 1999). The advancement of engineering tolerance to organic acids and phenolic compounds has been excellently summarized in recent reviews (Mills et al., 2009; Laluce et al., 2012). This review mainly focuses on furan aldehydes as important lignocellulose inhibitors.

**Figure 3:**
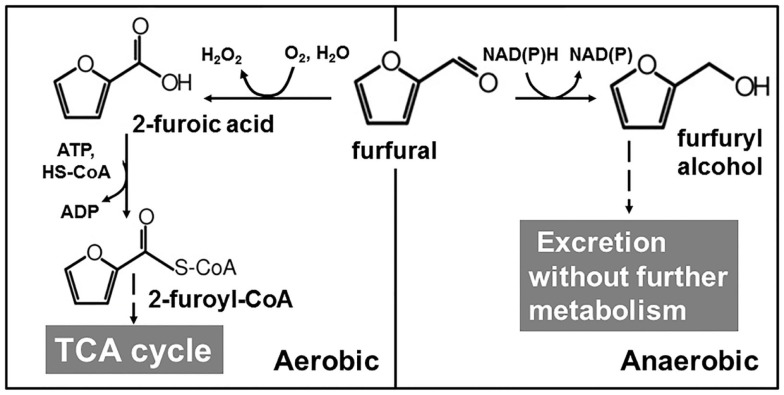
**Native furfural degradation pathways**. There are two major native metabolic routes for furfural. In some *Pseudomonas putida* strains, using oxygen as the final electron acceptor furfural goes through a series of oxidation and eventually goes into TCA cycle for further degradation. In contrast to aerobic degradation, the oxidoreductases with furfural reductase activity are recruited under anaerobic fermentation condition to reduce furfural to furfuryl alcohol, a less toxic product. Furfuryl alcohol is excreted into the medium.

A significant amount of effort has been contributed to the identification and optimization of biological components to increase the resistance to furan aldehydes, especially furfural (Table 1). The toxicity mode of furan aldehydes is complex and involves multiple factors (Almeida et al., 2009; Lin et al., 2009a,b; Mills et al., 2009). Cellular growth is arrested in the presence of furan aldehydes and growth resumes after the complete reduction of furfural. This furan-induced delay in growth was observed in both *E. coli* and *S. cerevisiae* (Taherzadeh et al., 2000; Miller et al., 2009b; Wang et al., 2012b). There are two major metabolic pathways to metabolize or reduce furan aldehydes in nature (Figure 3). Some bacteria such as *Cupriavidus basilensis* HMF14 can catabolize furan aldehyde as a sole carbon source when growing aerobically (Koopman et al., 2010). Furan aldehydes such as furfural are firstly oxidized into 2-furoic acid and then further metabolized to 2-oxoglutaric acid that eventually enters the TCA cycle to provide energy and biosynthetic building block (Trudgill, 1969; Koenig and Andreesen, 1990; Koopman et al., 2010) (Figure 3). The key step of this furfural degradation is dependent on oxygen thus limiting its application for anaerobic fermentative production (Koopman et al., 2010; Ran et al., 2014). *E. coli* and *S. cerevisiae* do not have furan aldehydes oxidative degradation pathways. Under anaerobic fermentation conditions, these microbes use their native oxidoreductases to reduce furan aldehydes to furan alcohol, which is much less toxic (Zaldivar et al., 1999, 2000). Furan alcohols are secreted outside of cells and remain in the fermentation broth without further degradation (Liu and Blaschek, 2010; Wang et al., 2012b). Cells do not grow until furfural or 5-HMF is reduced to a low threshold concentration (~5 mM) (Liu and Blaschek, 2010; Wang et al., 2012b; Ran et al., 2014) (Figure 3). This native detoxification approach has been strengthened in *S. cerevisiae* strains by overexpression of the native oxidoreducase genes such as *ADH1* (Laadan et al., 2008), *ADH6* (Petersson et al., 2006; Almeida et al., 2008; Liu et al., 2008), and *ADH7* (Liu et al., 2008) encoding the enzymes with activities to reduce furan aldehydes (Table 1). Overexpression of these oxidoreductase genes increases the 5-HMF reduction rate and shortens the lag time of cell growth. Interestingly, this native detoxification response causes the growth arrest in *E. coli*. The presence of furfural activates the expression of the *yqhD* gene encoding an oxidoreductase able to reduce furfural to furfuryl alcohol using NADPH as the reducing cofactor (Miller et al., 2009b; Turner et al., 2010). However, NADPH is essential for biosynthesis but is very limited under anaerobic xylose fermentation (Frick and Wittmann, 2005; Miller et al., 2009a). It is this depletion of NADPH by YqhD that has been proposed as the mechanism for growth inhibition in *E. coli* (Miller et al., 2009a,b; Turner et al., 2010). The NADPH-intensive pathway for sulfate assimilation was identified as a sensitive site that may be responsible for growth inhibition (Miller et al., 2009a). Addition of cysteine, deletion of *yqhD*, or increased expression of *pntAB* (transhydrogenase for interconversion of NADH and NADPH) conferred the tolerance to furan aldehydes including furfural and 5-HMF in *E. coli* (Miller et al., 2009a,b, 2010). To accelerate the furfural reduction but avoid using NADPH as the reducing cofactor, an alternative NADH-dependent furfural reductase is desired. A native oxidoreductase, FucO, was identified to have such properties and its overexpression did increase furfural tolerance in different *E. coli* biocatalysts (Wang et al., 2011). FucO normally functions in fucose metabolism and its catalytic efficiency for furfural reduction is low (Wang et al., 2011). The enzyme properties of FucO as a furfural reductase were improved by site-saturated mutagenesis and growth-based selection (Zheng et al., 2013). Overall, optimization of NADH-dependent furfural reductase has potential to shorten the lag phase and to increase tolerance of biocatalysts under fermentation conditions.

**Table 1:** **Beneficial genetic traits for furan aldehydes degradation and tolerance**.

Beneficial genetic traits	Microbial host	Proposed detoxification mechanism	Reference
*yqhD* deletion	*E. coli*	Avoid the competition for NADPH	Miller et al. (2009b)
*pntAB* overexpression	*E. coli*	Increase NADPH level	Miller et al. (2009a)
*fucO* overexpression	*E. coli*	Reduce furfural to furfuryl alcohol	Wang et al. (2011)
Mutation of *irrE*	*E. coli*	Stress related global regulator	Wang et al. (2012a)
*ucpA* overexpression	*E. coli*	Unknown	Wang et al. (2012b)
*thyA* overexpression	*E. coli*	Increase the availability of dTMP for DNA repair	Zheng et al. (2012)
*fucO* missense mutations	*E. coli*	Improve furfural reductase activity	Zheng et al. (2013)
*potE* overexpression	*E. coli*	Polyamine binding to negatively charged cellular constituents	Geddes et al. (2014)
*puuP* overexpression	*E. coli*	Polyamine binding to negatively charged cellular constituents	Geddes et al. (2014)
*plaP* overexpression	*E. coli*	Polyamine binding to negatively charged cellular constituents	Geddes et al. (2014)
*potABCD* overexpression	*E. coli*	Polyamine binding to negatively charged cellular constituents	Geddes et al. (2014)
*lpcA* overexpression	*E. coli*	Strengthen cell wall or indirectly increase NADPH availability	Glebes et al. (2014b)
*groESL* overexpression	*E. coli*	Possibly related to solvent stress response	Glebes et al. (2014b)
*ahpC* overexpression	*E. coli*	Unknown	Glebes et al. (2014a)
*yhiH* overexpression	*E. coli*	Unknown	Glebes et al. (2014a)
*rna* overexpression	*E. coli*	Unknown	Glebes et al. (2014a)
*dicA* overexpression	*E. coli*	Unknown	Glebes et al. (2014a)
*ZWF1* overexpression	*S. cerevisiae*	Maintain NADPH levels needed for furan oxidoreductases	Gorsich et al. (2006)
*ADH6* overexpression	*S. cerevisiae*	Reduce HMF to alcohol form	Petersson et al. (2006)
*ADH1* missense mutations	*S. cerevisiae*	S109P, L116S, and Y294C increase affinity to NADH	Almeida et al. (2008), Laadan et al. (2008)
*ADH7* overexpression	*S. cerevisiae*	Reduce HMF to alcohol form	Liu et al. (2008)
*YAP1* overexpression	*S. cerevisiae*	Mitigate oxidative stress	Ma and Liu, 2010, Kim and Hahn, 2013
Inactivation of *SIZ1*	*S. cerevisiae*	Likely related to oxidative stress	Xiao and Zhao, 2014
Aerobic HMF degradation	*C. basilensis HMF14*	Oxidize HMF	Koopman et al. (2010)
Aerobic furfural degradation	*C. basilensis HMF14*	Oxidize furfural	Trudgill, 1969, Koopman et al. (2010)
	*P. putida Fu1, C. basilensis HMF14*	

**Figure 4:**
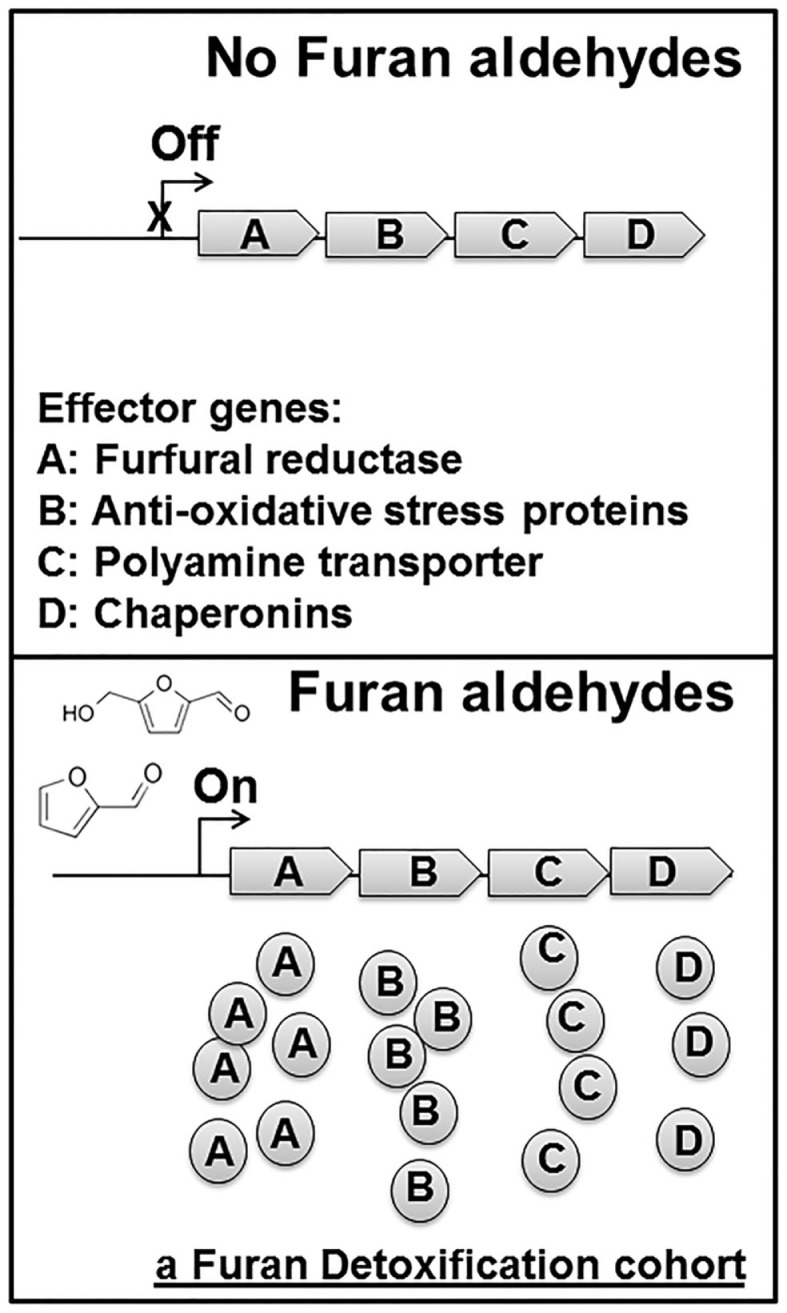
**The integrated furan aldehydes detoxification system**. A furfural responsive promoter and multiple effector genes are integrated into the chromosome. In the absence of furan aldehydes, this artificial operon is inactive and effector genes are not expressed. Furan aldehydes activate the responsive promoter to drive the expression of effector genes. Effectors are produced to mitigate the toxicity of furan aldehydes. Example effectors shown in the graph are furfural reductase **(A)**, anti-oxidative protein **(B)**, polyamine transporter **(C)**, and chaperonin **(D)**, assuming these effectors have synergistic epistatic interaction. When furfural level decreases, promoter remains silenced and no more new effectors are made. This design provides a controllable mechanism for furfural tolerance to minimize metabolic burden and maximize the benefit of effector genes.

A variety of genomic and transcriptomic approaches have yielded many beneficial genetic traits related to furan aldehydes tolerance (Table 1). *S. cerevisiae* gene disruption library was screened for mutants with growth deficiencies in the presence of furfural and *ZWF1* was found to relate to furfural tolerance (Gorsich et al., 2006). Overexpression of *ZWF1* increased furfural tolerance (Gorsich et al., 2006). *ZWF1* encodes glucose-6-phosphate dehydrogenase, which catalyzes the first step of the pentose phosphate pathway, the major pathway providing NADPH when utilizing glucose as the carbon source. A similar approach using genome-wide RNAi screen showed that inactivation of the *SIZ1* gene increased furfural tolerance (Xiao and Zhao, 2014). *SIZ1* encodes E3 SUMO-protein ligase and inactivation of *SIZ1* increases the tolerance to oxidative stress besides furfural (Xiao and Zhao, 2014). At least part of the toxicity mechanism induced by furfural is suggested to be associated with oxidative stress (Mills et al., 2009). Furfural was shown to induce the accumulation of reactive oxygen species inside of the *S. cerevisiae* cells and to cause damage to mitochondria, vacuole membranes, and cytoskeletons (Allen et al., 2010). Furan aldehydes were also reported to act as thiol-reactive electrophiles, to directly activate Yap1 transcription factor and to deplete glutathione (Kim and Hahn, 2013). Overexpression of either wild-type *YAP1* or its target genes *CTA1* and *CTT1*encoding catalases increased tolerance to furan aldehydes (Kim and Hahn, 2013). Interestingly, furan aldehydes do not induce oxidative responses in *E. coli*. The expression of the genes in major oxidative regulons such as OxyR and SoxRS regulons is not activated by the presence of furfural (Miller et al., 2009a). This strain difference adds another layer of complexity to engineering tolerance of furan aldehydes. In *E. coli*, an oxidoreductase UcpA with an undefined function was found to be associated with furfural tolerance by a transcriptomic analysis and its overexpression increased furan aldehyde tolerance (Wang et al., 2012b). Genomic libraries from three different bacteria were screened for genes that conferred furfural resistance to *E. coli* on plates. Beneficial plasmids containing the *thyA* gene were recovered from all three genomic libraries. The *thyA* gene encodes thymidylate synthase, important for dTMP biosynthesis, suggesting furfural toxicity is possibly related to DNA damage (Zheng et al., 2012). The microarray studies and whole genome sequencing of furfural resistant *E. coli* mutants led to the discovery of some polyamine transporters including PotE, PuuP, PlaP, and PotABCD with a beneficial role for furfural tolerance (Geddes et al., 2014). The detoxification mechanism was proposed to relate to the protection role of polyamine for important cellular constituents such as DNA (Geddes et al., 2014). Other advanced genomic tools such as multiSCale Analysis of Library Enrichments (SCALE) (Lynch et al., 2007) and trackable multiplex recombineering (TRMR) (Warner et al., 2010) have been used to identify more furfural related genetic traits in *E. coli* (Glebes et al., 2014a,b). These experiments showed the *lpcA*, *groESL*, *ahpC*, *yhiH*, *rna*, and *dicA* genes are associated with furfural tolerance although the overexpression of these genes individually only showed limited positive effect (Glebes et al., 2014a,b). Another interesting approach is to select a mutant form of the stress-related exogenous regulator IrrE, which confers *E. coli* the tolerance to furan aldehydes (Wang et al., 2012a). Considering the complexity of the toxicity mode induced by furfural, it is not surprising to identify multiple biological parts beneficial for furan tolerance (Table 1). However, all these individual beneficial genetic traits discussed above only provide limited improvement for furan aldehyde tolerance. How to combine multiple beneficial genetic traits to achieve a significant increase of tolerance is a great challenge for synthetic biologists. An ideal synthetic detoxification system should contain a furfural responsive promoter driving the expression of the optimal combinations of different effector genes to minimize metabolic burden and maximize the benefit of effector genes (Figure 4).

There are at least two major challenges for designing such an integrated detoxification system. First, most epistatic interactions between beneficial genetic traits are not predictable and the experimental search for the optimal combination of multiple effector genes is time-consuming and labor-intensive (Sandoval et al., 2012b). Negative epistatic interactions are present for different beneficial genetic traits for furan aldehyde tolerance. For example, the combination of two beneficial traits, the increased expression of *pntAB* and the deletion of the *yqhD* gene together, made cells less tolerance to furfural than the cells with either one of these two beneficial genetic traits alone (Wang et al., 2013). Further characterization of the beneficial traits in a high-throughput manner is desired to eventually construct an optimal combination of multiple effector genes. Second, the technical challenges to achieve optimal expression of effector genes at the chromosomal level remain to be solved. The effector genes are normally expressed from an expression vector with expensive inducers and antibiotics or other selective conditions. The application of a plasmid-based expression system is undesired in large-scale bio-based production conditions due to the genetic instability, metabolic burden, and the costs (Keasling, 2008; Jarboe et al., 2010). Integration of furan aldehydes detoxification systems into the chromosome is desired. However, it is challenging to achieve the optimal expression of target genes at the chromosomal level, especially when high expression is needed.

## Conclusion and Future Perspectives

Efficient xylose metabolism and tolerance to furan aldehydes are desired features of microbial catalysts used in lignocellulose conversion. Past efforts of synthetic biology focused on identification and optimization of individual biological parts needed for a successful lignocellulose conversion. We have gradually accumulated much knowledge about xylose metabolism and transport, glucose repression, and furan aldehyde toxicity. Limited success of lignocellulose conversion has been achieved using these individual optimized parts (Sandoval et al., 2012a; Wang et al., 2013). Instead of taking a reductionist approach, we are reaching a new phase to characterize the epistatic interactions and to integrate the optimal combinations of different biological parts. This development is dependent on the modular high-throughput approach for epistasis characterization and large-scale genome editing. With the new development of high-throughput techniques and genome editing tools such as CRISPR/Cas9 technology (Doench et al., 2014; Harrison et al., 2014; Sampson and Weiss, 2014), constructing an effective platform strain for lignocellulose conversion is in the scope. The platform strains with high efficiency of sugar co-utilization and tolerance to chemical insult can be used to produce a variety of fuels and chemicals from lignocellulosic biomass by metabolic engineering. These common platforms can also be tuned to different types of biomass by laboratory adaptive evolution.

## Conflict of Interest Statement

The authors declare that the research was conducted in the absence of any commercial or financial relationships that could be construed as a potential conflict of interest.
